# Orange juice–derived flavanone and phenolic metabolites do not acutely affect cardiovascular risk biomarkers: a randomized, placebo-controlled, crossover trial in men at moderate risk of cardiovascular disease^1^,^2^,^3^,^4^,^5^

**DOI:** 10.3945/ajcn.114.104364

**Published:** 2015-03-18

**Authors:** Manuel Y Schär, Peter J Curtis, Sara Hazim, Luisa M Ostertag, Colin D Kay, John F Potter, Aedín Cassidy

**Affiliations:** 1 From the Department of Nutrition, Norwich Medical School, University of East Anglia, Norwich, United Kingdom.

**Keywords:** flavonoids, flavanones, phenolic metabolites, randomized controlled trial, vascular function

## Abstract

**Background:** Epidemiologic data suggest inverse associations between citrus flavanone intake and cardiovascular disease (CVD) risk. However, insufficient randomized controlled trial data limit our understanding of the mechanisms by which flavanones and their metabolites potentially reduce cardiovascular risk factors.

**Objective:** We examined the effects of orange juice or a dose-matched hesperidin supplement on plasma concentrations of established and novel flavanone metabolites and their effects on cardiovascular risk biomarkers in men at moderate CVD risk.

**Design:** In an acute, randomized, placebo-controlled crossover trial, 16 fasted participants (aged 51–69 y) received orange juice or a hesperidin supplement (both providing 320 mg hesperidin) or control (all matched for sugar and vitamin C content). At baseline and 5 h postintake, endothelial function (primary outcome), blood pressure, arterial stiffness, cardiac autonomic function, platelet activation, and NADPH oxidase gene expression and plasma flavanone metabolites were assessed. Before each intervention, a diet low in flavonoids, nitrate/nitrite, alcohol, and caffeine was followed, and a standardized low-flavonoid evening meal was consumed.

**Results:** Orange juice intake significantly elevated mean ± SEM plasma concentrations of 8 flavanone (1.75 ± 0.35 μmol/L, *P* < 0.0001) and 15 phenolic (13.27 ± 2.22 μmol/L, *P* < 0.0001) metabolites compared with control at 5 h postconsumption. Despite increased plasma flavanone and phenolic metabolite concentrations, cardiovascular risk biomarkers were unaltered. After hesperidin supplement intake, flavanone metabolites were not different from the control, suggesting altered absorption/metabolism compared with the orange juice matrix.

**Conclusions:** After single-dose flavanone intake within orange juice, circulating flavanone and phenolic metabolites collectively reached a concentration of 15.20 ± 2.15 μmol/L, but no effects were observed on cardiovascular risk biomarkers. Longer-duration randomized controlled trials are required to examine previous associations between higher flavanone intakes and improved cardiovascular health and to ascertain the relative importance of food matrix and flavanone-derived phenolic metabolites. This trial was registered at clinicaltrials.gov as NCT01530893.

See corresponding editorial on page 897.

## INTRODUCTION

Cardiovascular disease (CVD) is a major cause of mortality worldwide, accounting for 32% of deaths in the United States and United Kingdom, with men being at a higher risk than women (1, 2). Recent data suggest that diets rich in fruit were the third most important modifiable factor for reducing global CVD-related morbidity and mortality, with only blood pressure–lowering medication and smoking cessation having greater effects (3). As citrus fruit is consumed widely, the cardiovascular protective effects of fruit per se may in part be related to citrus fruit intake and specifically to their flavanone (a flavonoid subclass) and vitamin C content (4). In support of this, epidemiologic data provide evidence to suggest that flavanones and intake of citrus fruit are inversely associated with CVD mortality (5–7). In addition, animal and in vitro studies have reported that flavanones improve endothelial function [principally via enhanced production or reduced NADPH oxidase-dependent elimination of endothelial nitric oxide (8, 9)] and cardiac autonomic function (10), as well as reduce blood pressure (11) and platelet reactivity (12). However, there remain insufficient data from human randomized controlled trials to determine the relative bioactivity of dietary flavanones and their metabolites. Moreover, acute and chronic studies are needed to establish the relative effects of the different bioactive constituents, particularly the flavanones and vitamin C.

In the limited number of previously published randomized controlled trials, improvements in endothelial function or a reduction in diastolic blood pressure have been observed after short-chronic intake (3- or 4-wk duration) of a flavanone supplement and/or orange juice (9, 13). However, few studies to date have established acute responses to flavanones, and thus the potential mechanisms for a cardiovascular benefit remain unclear. Although improvements in endothelium-dependent microvascular reactivity have been observed after acute flavanone supplement and orange juice intake (13), currently no data support the acute effect of dietary flavanones on central arterial stiffness, cardiac autonomic regulation, or platelet reactivity.

A growing number of bioavailability studies have established the pharmacokinetic characteristics of a range of flavanone metabolites (i.e., phase II conjugates of the parent structure) after orange juice intake (14–16), with data suggesting that the phenolic metabolites contribute substantially to flavanone bioavailability (17, 18). However, few studies have examined the potential importance of orange juice–derived flavanone and phenolic metabolites on cardiovascular function. Studies investigating similar phenolic metabolites derived from olive oil or blueberries have, however, reported acute effects on cardiovascular risk biomarkers (19, 20).

We therefore conducted a 3-arm, acute, randomized, placebo-controlled crossover trial in men at moderate CVD risk, in whom endothelial function, a range of cardiovascular risk biomarkers, and plasma flavanone metabolites were assessed 5 h after ingestion of a single dose of orange juice, a hesperidin- and vitamin C–matched supplement, or a matched control.

## METHODS

### Study population

Sixteen healthy men aged 51–69 y, who were nonsmokers but with a 10–20% risk of CVD in the following 10 y [estimated by using the British Hypertension Society absolute risk calculator (21)] at screening, were recruited and enrolled by the research team (comprising research scientists and research nurses). Ineligibility criteria were past or existing diabetes; cancer or hepatic, renal, digestive, hematologic, neurologic, or thyroidal diseases; a resting blood pressure >160/95 mm Hg at screening; or use of lipid-lowering, antihypertensive, or vasoactive medication. In addition, antibiotic or flu vaccination (within 3 mo before and during the study) was precluded, given the impact of antibiotics on microflora and flavanone bioavailability (22), as was intake of flavonoid-containing supplements (for ≥1 mo before and throughout the study). Clinical guidance was sought when changes in health were reported, and continued eligibility to participate was monitored. The study was approved by the local ethics committee; followed the principles of the Declaration of Helsinki; was conducted at the Clinical Research and Trials Unit, University of East Anglia (United Kingdom) between April 2012 and May 2013; and was registered at clinicaltrials.gov as NCT01530893. All participants provided written informed consent before study commencement.

### Study design

Before identical experimental periods (≥1 wk apart), participants followed a diet low in flavonoids (for 72 h); refrained from strenuous exercise (for 48 h); avoided alcohol, caffeine, and nitrate/nitrite-containing foods (for 24 h); and drank only commercially available bottled water low in potentially vasoactive nitrate/nitrite (Buxton Water) (for 24 h). The night before each experimental period, participants consumed a standardized low-flavonoid meal (<1 mg estimated flavonoid content) before observing an overnight fast (with only bottled water consumed for ≥10 h). Compliance to the diet and lifestyle restrictions were assessed at interview, and adherence to the dietary restrictions was determined via a 24 h-dietary recall. Food intake data were assessed by using a standardized food intake database (WISP V4.0; Tinuviel Software).

Experimental periods were conducted in a quiet, light-subdued, and temperature-ambient (22–24°C) room with cardiovascular measures performed on the same body side on each occasion in a supine position at rest (≥15 min) and following standardized procedures. Three blood pressure assessments were made by using a validated monitor (Omron 705IT; Omron Health Care Co.). Cardiac baroreflex sensitivity (a measure of cardiac autonomic function) was assessed from 12 min of continuous middle-finger arterial pressure data (by using a Portapres device operated by Beatscope 1.1. software with hand-to-heart height adjustment; TNO Biomedical Instrumentation) and analyzed by the sequential cross-correlation method (23). Reactive hyperemia-peripheral arterial tonometry (RH-PAT), a measure of endothelial function in the microvasculature of the finger, was assessed by using EndoPAT equipment (Itamar Medical) as previously described (24). Carotid-to-femoral pulse wave velocity and central augmentation index assessment (normalized for heart rate at 75 beats/min), measures of central arterial stiffness, were assessed by using Vicorder equipment (Skidmore Medical) as described previously [(25) and (26), respectively]. The central augmentation index assessment was normalized for heart rate because of the known association between the augmentation index and heart rate (27). Blood samples were collected at the midpoint of the battery of cardiovascular measures on the contralateral arm, immediately processed, and stored at −80°C. Height and weight were measured to calculate BMI (in kg/m^2^).

The intervention was administered after baseline assessment, and cardiovascular measures and blood collection were repeated in an identical sequence at 5 h postintervention; 5 h was selected to coincide with the anticipated time of maximal flavanone plasma concentration, on the basis of previous data (15). During the experimental period, participants consumed low-nitrate/nitrite water ad libitum, and their diet was controlled: a flavonoid-free standardized lunch (660 kcal, 31.1 g protein, 95.6 g carbohydrate, and 26.8 g fat) was provided 1.5 h after the intervention.

### Intervention products

Before study commencement, orange juice (supplied by the Florida Department of Citrus) and hesperidin supplement (a kind gift from Miguel Aragüés, Alicante, Spain) were analyzed for hesperidin, narirutin, and vitamin C content by using HPLC-diode array detection (14) (typical chromatograms are shown in **Supplemental Figure 1**). A dose of 320 mg hesperidin, chosen on the basis of vascular efficacy (13) and achievable dietary intakes (6), was provided by 767 mL orange juice (**Table 1**) that was sourced from a single batch and stored at −20°C. The hesperidin supplement intervention was matched in hesperidin content, whereas all interventions (i.e., orange juice, hesperidin supplement, and control) were matched for vitamin C, sugars, and fluid volume by using a low-nitrate/nitrite bottled water, given the potential vasoactive properties of nitrate/nitrite-containing products (28) (Table 1). At each experimental period, intervention drinks (orange juice or control) were prepared by independent scientists (following a computer-generated, random-sequence allocation, held by an independent scientist) and served in opaque drinking vessels. Scientists conducting the research and analyzing the data remained blinded throughout the study, whereas participants were blinded to the hesperidin supplement but not the orange juice intervention because of unavoidable differences in taste. Flavanone contents of the orange juice and hesperidin supplement were stable over the course of the study (pre- and poststudy CV = 5.0%).

**TABLE 1:** Composition of the intervention products

	Intervention		
	Control	Orange juice	Hesperidin supplement
Drink volume, mL	767	767	767
Supplement	Vitamin C	Cellulose	Hesperidin supplement and vitamin C
Sugar,^1^ g	68.0	68.0	68.0
Hesperidin,^2^ mg	0	320	320
Narirutin,^2^ mg	0	48	16
Vitamin C,^2^ mg	439	439	439

1 Matched sugar composition: 16.6 g glucose, 18.0 g fructose, and 33.4 g sucrose (data provided by the Florida Department of Citrus).

2 Quantified in house by using HPLC.

### Plasma metabolite analysis

Naringenin-7-glucuronide was purchased from Bioquote, 3-hydroxyhippuric acid from Enamine, and hesperetin, naringenin, and 53 phenolics from Sigma Aldrich (for the list of all phenolics, see **Supplemental Figure 2**). Glucuronide or sulfate conjugates of protocatechuic acid, vanillic acid, isovanillic acid, and benzoic acid were synthesized at the University of St. Andrew following published methods (29). After solid-phase extraction (using Strata-X columns; Phenomenex), acidified heparinized plasma was analyzed by HPLC (1200 Agilent series HPLC; Agilent Technologies) coupled to an electrospray ionization mass spectrometer (AB Sciex 3200 series Q-trap MS/MS; AB Sciex) in scheduled multiple-reaction monitoring mode and by using previously optimized and validated methods (30), with modifications for the quantification of flavanone phase II metabolites [based on previous findings (14, 15, 17)] and putative phenolic phase II metabolites, of which analytic standards were not available. A systematic screen of a pooled plasma sample was explored for 158 potential metabolites. For a description of the screening strategy, a typical HPLC–mass spectrometer trace of the final method, the corresponding analytic identification parameters, and compound structures with Biochemical Nomenclature, see Supplemental Figure 2, **Supplemental Figure 3**, **Supplemental Table 1**, and **Supplemental Table 2**, respectively.

Plasma vitamin C was analyzed by using commercial ascorbate assay kits (No. 7004240; Cayman Chemical).

### Plasma cardiovascular risk biomarkers

Platelet reactivity was assessed by whole-blood flow cytometry as previously described (31). Briefly, sodium-citrated blood was treated with fluorophore-conjugated monoclonal antibodies (i.e., CD61-allophycocyanin and CD62p-phycoerythrin from eBioscience and PAC1-fluorescein isothiocyanate from BD bioscience), and platelets were left unstimulated or activation was induced by addition of ADP (1 μmol/L; Alpha Laboratories) or collagen-related peptide (0.4 μg/mL; Department of Biochemistry, University of Cambridge). After a 20-min incubation in the dark at room temperature, samples were fixed with 1% formyl saline and stored at 4°C in the dark until analysis on the same day by using a BD Accuri C6 Flow Cytometer (Becton Dickinson). Ten thousand platelet events (i.e., events with CD61-positive fluorescence) were collected and platelet reactivity was assessed as the percentage of platelets that expressed P-selectin (i.e., CD62p-positive fluorescence) and the activated conformation of fibrinogen-binding receptor glycoprotein IIb/IIIa (i.e., PAC1-positive fluorescence).

NAPDH oxidase gene expression was assessed by measuring serum soluble gp^91phox^ (i.e., the catalytic core of NADPH oxidase) by using ELISA as previously described (32). Briefly, duplicate serum samples were assayed with anti-gp91^phox^ (Santa Cruz Biotechnology) coated plates, and spectrophotometric quantification was established against standard curves (New England Peptide).

### Sample size and statistical analysis

To detect a 0.35 increase in RH-PAT (our primary outcome measure) [assuming an SD of 0.4, based on previous data (33)] with 80% power and at the 5% significance level, we required a sample size of 14 participants to complete the study. Differences in study endpoints between interventions were analyzed by using a linear mixed model for crossover studies (34), with subjects nested within intervention sequence as a random effect and experimental period, intervention sequence, baseline values, and intervention as fixed effects. No carryover effects were observed, as assessed by the experimental period and intervention interaction (*P* > 0.05). When the model showed a significant intervention effect, pairwise comparisons between interventions were performed with Tukey-Kramer adjustments. Associations between cardiovascular risk biomarkers and plasma concentrations of total flavanone or total phenolic metabolites were assessed by Pearson’s correlation. Data are presented as means ± SEMs. *P* values <0.05 were considered statistically significant, and statistical analysis was performed by using R programming language version 3.1.1 (R Development Core Team, 2009).

## RESULTS

**FIGURE 1:**
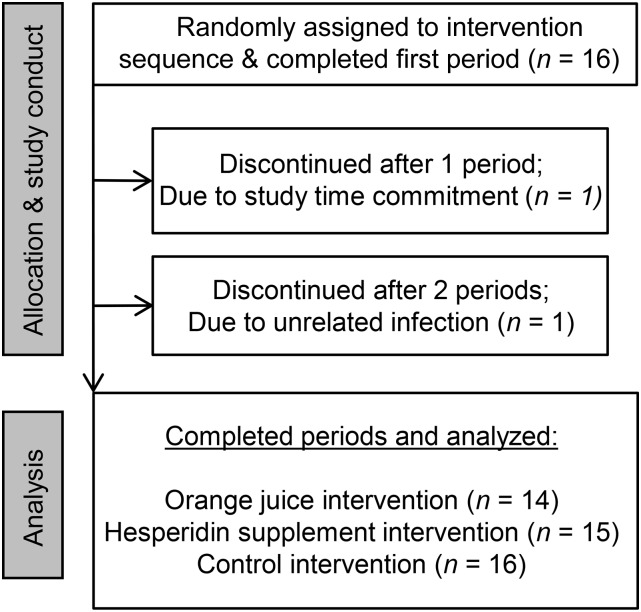
Participant flowchart.

**FIGURE 2:**
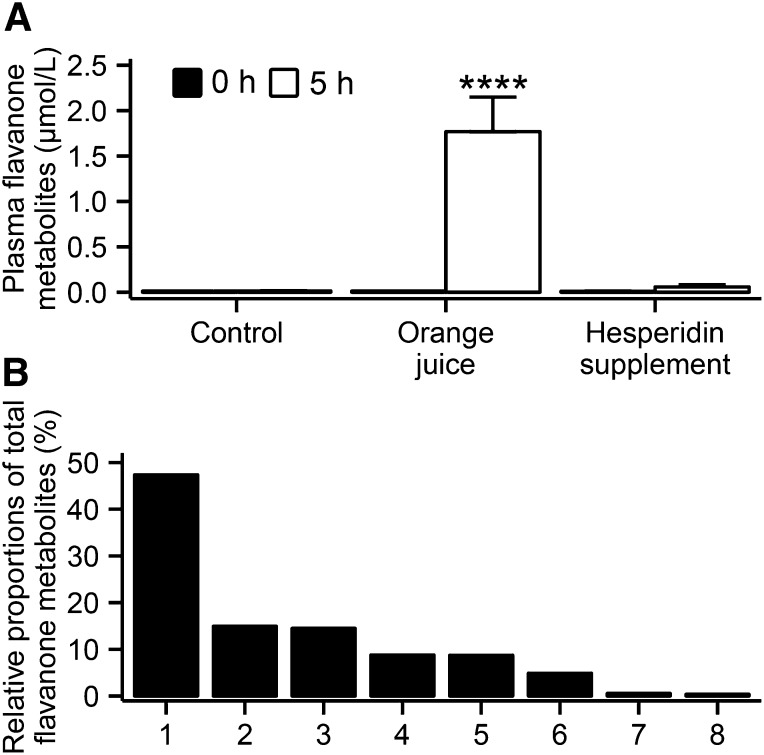
Mean ± SEM plasma concentrations of total flavanone metabolites (A) at baseline (i.e., 0 h) and 5 h after intervention in men at moderate cardiovascular disease risk [*n* = 16 (control), *n* = 14 (orange juice), and *n* = 15 (hesperidin supplement)]. Differences in study endpoints between interventions were analyzed by using a linear mixed model for crossover studies, with subjects nested within the intervention sequence as a random effect and experimental period, intervention sequence, baseline values, and intervention as fixed effects (intervention effect: *P* < 0.0001). Post hoc analysis with Tukey-Kramer adjustment was performed to determine which intervention was significantly different from control. ****P < 0.0001. Identified flavanone metabolites (B) and their proportions relative to total flavanone metabolite plasma concentrations at 5 h after the orange juice intervention: 1, hesperetin-glucuronide; 2, naringenin-7-glucuronide; 3, hesperetin-glucuronide; 4, hesperetin-diglucuronide; 5, hesperetin-diglucuronide; 6, naringenin-glucuronide; 7, hesperetin; and 8, naringenin.

Study participants were healthy men, with a mean age of 61 y (range: 51–69 y) and an estimated 15.8% (range: 10–20%) absolute risk of CVD in the following 10 y (**Table 2**). Among the 16 enrolled participants, 14 completed all arms of the study; trial participation was discontinued by 1 participant (after 1 experimental period) because of inability to meet the study time commitments, and 1 participant withdrew after 2 experimental periods after developing an unrelated infection (**Figure 1**). No serious adverse events were reported. All participants reported adhering to dietary and exercise restrictions, and low plasma concentrations of flavanone metabolites at baseline confirmed adherence to a flavanone-free diet (**Figure 2A**). Estimated habitual energy intakes before the study day and BMI were similar between the intervention periods (data not shown), as were baseline measures of cardiovascular risk biomarkers (**Table 3**).

**TABLE 2:** Characteristics of overnight-fasted study participants (*n* = 16) at screening

	Mean ± SEM	Range
Age, y	60.6 ± 1.4	51–69
Absolute cardiovascular disease risk,^1^ %	15.8 ± 0.7	10–20
BMI, kg/m^2^	25.6 ± 0.8	22–35
Systolic blood pressure, mm Hg	136.2 ± 3.0	111–155
Diastolic blood pressure, mm Hg	82.6 ± 1.6	73–94
Plasma glucose, mmol/L	5.0 ± 0.1	3.9–6.0
Plasma triglycerides, mmol/L	1.1 ± 0.1	0.6–2.3
Plasma total cholesterol, mmol/L	5.2 ± 0.1	4.2–6.5
Plasma HDL cholesterol, mmol/L	1.3 ± 0.1	0.9–1.9
Plasma LDL cholesterol, mmol/L	3.4 ± 0.1	2.6–4.4

1 Ten-year absolute cardiovascular disease risk as calculated by using the British Hypertension Society risk calculator (21).

**TABLE 3:** Cardiovascular risk biomarkers at baseline and 5 h after orange juice, hesperidin supplement, or control intervention in men at moderate cardiovascular disease risk^1^

	Control			Orange juice			Hesperidin supplement			
	BL	5 h	Δ	BL	5 h	Δ	BL	5 h	Δ	*P*^2^
Blood pressure, mm Hg										
Systolic	128.2 ± 2.2	123.6 ± 1.8	−4.7 ± 2.6	126.3 ± 1.8	123.6 ± 2.9	−2.6 ± 2.2	127.1 ± 2.0	123.9 ± 2.5	−3.2 ± 1.9	0.87
Diastolic	80.2 ± 1.6	75.1 ± 1.8	−5.1 ± 1.2	77.9 ± 1.8	73.6 ± 1.9	−4.2 ± 1.2	80.8 ± 1.9	75.6 ± 1.8	−5.2 ± 1.1	0.91
Heart rate, beats/min	54.9 ± 2.0	58.6 ± 2.2	3.7 ± 1.2	53.9 ± 1.9	58.4 ± 2.2	4.4 ± 1.1	54.5 ± 2.1	58.3 ± 2.0	3.7 ± 1.2	0.74
RH-PAT index	2.78 ± 0.18	2.66 ± 0.17	−0.12 ± 0.09	2.77 ± 0.13	2.68 ± 0.19	−0.09 ± 0.11	2.85 ± 0.21	2.69 ± 0.15	−0.16 ± 0.14	0.96
Cardiac BRS, ms/mm Hg	11.2 ± 2.5	10.1 ± 1.3	−1.1 ± 1.5	11.2 ± 1.6	9.4 ± 1.0	−1.8 ± 1.1	10.9 ± 1.4	9.7 ± 1.0	−1.2 ± 1.1	0.58
cfPWV, m/s	9.7 ± 0.4	9.8 ± 0.3	0.1 ± 0.2	9.9 ± 0.3	9.8 ± 0.3	−0.1 ± 0.3	9.6 ± 0.5	9.9 ± 0.4	0.2 ± 0.3	0.77
cAIx@HR75, %	34.4 ± 2.1	28.2 ± 2.0	−6.1 ± 1.0	35.5 ± 2.4	30.2 ± 1.7	−5.2 ± 1.2	35.0 ± 2.1	28.7 ± 1.7	−6.3 ± 1.7	0.39
Serum soluble gp91^phox^, pg/mL	48.9 ± 5.2	48.4 ± 3.6	−0.5 ± 5.3	48.6 ± 5.9	51.7 ± 5.9	3.1 ± 4.9	58.3 ± 4.6	48.5 ± 5.5	−9.8 ± 6.7	0.67
Plasma vitamin C, μmol/L	33.1 ± 2.9	51.8 ± 3.7	18.7 ± 2.6	31.9 ± 3.6	47.9 ± 4.7	16.0 ± 2.2	31.2 ± 2.4	52.6 ± 5.3	21.4 ± 3.7	0.29
P-selectin expression, %										
Unstimulated platelets	3.6 ± 1.1	2.1 ± 0.6	−1.5 ± 0.5	4.5 ± 1.0	2.9 ± 0.8	−1.6 ± 0.4	3.6 ± 0.9	2.9 ± 0.7	−0.7 ± 0.6	0.18
ADP-activated platelets	95.8 ± 0.8	93.8 ± 1.3	−2.0 ± 0.6	96.2 ± 0.8	94.2 ± 1.2	−2.0 ± 1.1	94.8 ± 1.1	94.8 ± 0.8	0.0 ± 0.7	0.14
Collagen-related peptide-activated platelets	87.5 ± 3.8	88.8 ± 3.4	1.3 ± 1.8	87.2 ± 3.7	86.3 ± 4.0	−0.9 ± 1.5	87.6 ± 3.7	86.0 ± 3.9	−1.6 ± 0.9	0.28
Fibrinogen receptor expression, %										
Unstimulated platelets	1.5 ± 0.4	1.6 ± 0.5	0.1 ± 0.4	1.4 ± 0.3	1.1 ± 0.2	−0.3 ± 0.1	1.3 ± 0.3	1.6 ± 0.4	0.2 ± 0.4	0.50
ADP-activated platelets	77.6 ± 3.0	74.6 ± 3.3	−3.0 ± 1.4	78.4 ± 2.9	74.6 ± 2.8	−3.8 ± 2.2	75.6 ± 3.3	75.6 ± 2.9	0.0 ± 1.3	0.18
Collagen-related peptide-activated platelets	81.1 ± 4.6	85.0 ± 2.8	3.8 ± 3.0	80.2 ± 4.3	80.5 ± 4.3	0.3 ± 1.4	81.3 ± 4.3	80.9 ± 4.1	−0.4 ± 1.3	0.18

1 Values are means ± SEMs. Numbers for the interventions were *n* = 16 (control), *n* = 14 (orange juice), and *n* = 15 (hesperidin supplement), except for cfPWV and cAIx@HR75 [*n* = 15 (control), *n* = 13 (orange juice), and *n* = 14 (hesperidin supplement); *n* = 1 participant was excluded because of poor-quality tracings] and for platelet activation [*n* = 13 (control; *n* = 3 were excluded because of hemolyzed samples), *n* = 13 (orange juice; *n* = 1 was excluded because of hemolyzed samples), and *n* = 15 (hesperidin supplement)]. BL, baseline; BRS, baroreflex sensitivity; cAIx@HR75, central augmentation index corrected for heart rate; cfPWV, carotid to femoral pulse wave velocity; gp91^phox^, catalytic core of NADPH oxidase; RH-PAT, reactive hyperemia-peripheral arterial tonometry; Δ, 5-h postintervention changes from baseline.

2 Differences in study endpoints between interventions were analyzed by using a linear mixed model for crossover studies, with subjects nested within intervention sequence as a random effect and experimental period, intervention sequence, baseline values, and intervention as fixed effects. When the model showed a significant intervention effect, pairwise comparisons between interventions were performed with Tukey-Kramer adjustments, and changes were considered significant at *P* < 0.05.

**FIGURE 3:**
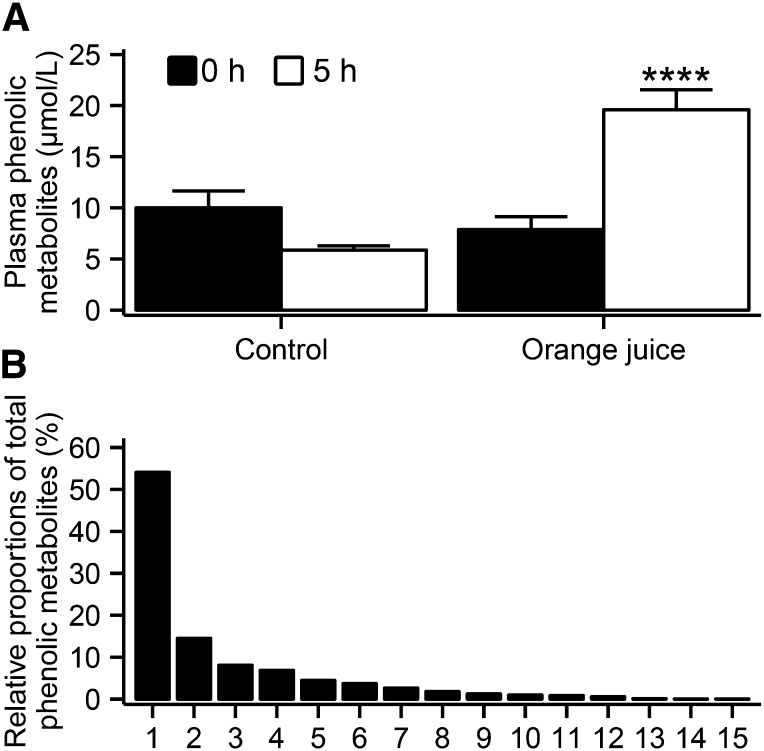
Mean ± SEM plasma concentrations of total phenolic metabolites (A) at baseline (i.e., 0 h) and 5 h after the control (*n* = 16) and orange juice (*n* = 14) intervention in men at moderate cardiovascular disease risk. Differences in study endpoints between interventions were analyzed by using a linear mixed model for crossover studies, with subjects nested within intervention sequence as a random effect and experimental period, intervention sequence, baseline values, and intervention as fixed effects (intervention effect: ****P < 0.0001). Identified phenolic metabolites (B) and their proportions relative to total phenolic metabolite plasma concentrations at 5 h after the orange juice intervention: 1, hippuric acid; 2, dihydroferulic acid; 3, dihydroferulic acid–3-glucuronide; 4, 4-hydroxyphenylacetic acid; 5, vanillic acid; 6, hydroxyhippuric acid; 7, iso/ferulic acid–glucuronide; 8, 3-hydroxyhippuric acid; 9, isovanillic acid; 10, 3-hydroxyphenylacetic acid; 11, vanillic acid–glucuronide; 12, isovanillic acid–glucuronide; 13, iso/vanillic acid–glucuronide; 14, 4-hydroxy-benzoic acid; and 15, benzoic acid–4-glucuronide.

Total plasma flavanone metabolite concentrations were significantly higher 5 h after the orange juice intervention than after control (orange juice elevation vs. control: 1.75 ± 0.35 μmol/L) (Figure 2A), with hesperidin-glucuronide, naringenin-7-O-glucuronide, and a second hesperidin-glucuronide contributing 47%, 15%, and 14%, respectively, to the total plasma flavanone concentration (Figure 2B). The concentration of total phenolics was significantly higher 5 h after orange juice ingestion than after control (orange juice elevation vs. control: 13.27 ± 2.22 μmol/L) (**Figure 3A**), and concentrations were 8-fold higher than the concentrations of total flavanones quantified. The major phenolic metabolites were hippuric acid, dihydroferulic acid, dihydroferulic acid–glucuronide, 4-hydroxyphenylacetic acid, and vanillic acid, contributing 54%, 15%, 8%, 7%, and 5%, respectively, to the total plasma phenolic metabolite concentration (Figure 3B). Unexpectedly, concentrations of plasma flavanone metabolites were not significantly elevated 5 h after the hesperidin supplement intervention compared with control (*P* = 0.9) (Figure 2A). Plasma vitamin C concentrations increased to a similar extent 5 h after ingestion of all interventions (Table 3).

Across the cardiovascular risk biomarkers that were assessed (blood pressure, endothelial function, central arterial stiffness, cardiac autonomic function, platelet activation, and NADPH oxidase gene expression), no significant differences were observed between the orange juice, hesperidin supplement, and control interventions (Table 3). Likewise, the concentration of total flavanone or total phenolic metabolites at 5 h after the orange juice intervention were not significantly associated with cardiovascular risk biomarkers (data not shown).

## DISCUSSION

In this acute intervention study, we examined whether the consumption of orange juice or a hesperidin supplement, matched for vitamin C, would result in acute effects on endothelial function and a number of previously unassessed cardiovascular risk biomarkers, including central arterial stiffness (assessed by central pulse wave velocity and augmentation index), cardiac baroreflex sensitivity, platelet reactivity, and NADPH oxidase gene expression. In addition, we explored associations between plasma flavanone or phenolic metabolites and cardiovascular risk biomarkers.

We observed no acute change in cardiovascular risk biomarkers, including endothelial function, 5 h after orange juice or hesperidin consumption (Table 3). These findings therefore do not support the previously reported acute benefits of orange juice or flavanone supplementation on peripheral microvascular reactivity (13). However, this study differed significantly in the methods used to assess microvascular reactivity, times of endpoint analysis, and, notably, the design of control arms. Specifically, in the present study, vitamin C was controlled across all interventions (including control), whereas in the study by Morand et al. (13), vitamin C was not present in the flavanone and control intervention arms. Indeed, in the present study, there was a tendency toward favorable changes in a number of vascular function markers (including diastolic blood pressure, endothelial function, and central augmentation index; Table 3) in all groups (including the vitamin C–matched control). This observation suggests that vitamin C may be an important contributor to the bioactivity of orange juice. It has also been shown that measurements of vascular reactivity vary between different assessment methods (35), likely because of differences in the vascular beds being assessed, which may further explain the differential findings between this and previous studies of microvascular reactivity.

In the present study, we identified an extensive number of plasma flavanone and phenolic metabolites, including 2 flavanone aglycones, 6 flavanone-glucuronides (as previously identified) (14–16), and a range of circulating phenolic metabolites after orange juice intake, of which 7 were previously detected only in urine (17, 18) and 8 previously unidentified (Figures 2B and  3B). Total plasma flavanone conjugate concentration (Figure 2A) reflected those previously reported in the literature (i.e., 0.2–1.6 μmol/L) (14, 15, 17), whereas total phenolic metabolite concentrations (Figure 3A) were observed at concentrations 8-fold higher than those of the flavanone metabolites (Figure 2A). This finding is further supported by studies that reported the recovery of 37–88% of ingested flavanones as phenolic metabolites in 24-h urine after intake of orange juice (17, 18). In the present study, we also report previously unidentified phenolic metabolites, again highlighting the complexity of flavanone metabolism. Overall, these data indicate that after flavanone consumption, the major circulating metabolites are phenolic breakdown products derived from the flavonoid backbone.

Although our data did not support correlations between cardiovascular biomarkers and the concentration of flavanone or phenolic metabolites, plasma hesperetin metabolites (phase II metabolites of the parent structure) have been associated with beneficial effects on endothelial function after acute flavanone intake in previous studies (*r* = 0.70, *P* = 0.0001) (13). Our data have shown that phenolic metabolites of flavanones are abundant in plasma within 5 h of orange juice intake, and these metabolites should remain a focus of future research exploring their contribution to the vascular effects of orange juice. Interestingly, the collective plasma concentration of the 23 metabolites was comparable to the observed increase in concentrations of plasma vitamin C (Table 3), suggesting that they collectively reach concentrations of potential biological significance (i.e., 15.20 ± 2.15 μmol/L). In contrast to the orange juice intervention, there were minimal flavanone and phenolic metabolites identified 5 h after intake of the hesperidin supplement. Interestingly, the bioavailability of another flavonoid, quercetin, and its monomethylated derivatives (isorhamnetin and tamarixetin) also has been shown to differ substantially depending on the dietary source. Plasma concentrations were 3.5–9 times lower when quercetin was consumed as a supplement than with a matched intake within a food matrix (36).

A number of limitations of the present study are worth noting. Although cardiovascular risk biomarkers were assessed at 5 h after intake, which is the anticipated peak flavanone plasma concentration [based on previous flavanone pharmacokinetic data (15)], it remains possible that beneficial cardiovascular effects could have been observed after a more prolonged (5–24 h or several weeks/mo) exposure. In addition, the present study used the RH-PAT index to measure fingertip endothelial function, and although flow-mediated dilatation of the brachial artery is generally accepted as a more sensitive method of assessing endothelial function (37) and CVD risk (38), an improvement in the RH-PAT index has recently been observed after an acute flavonoid intervention (39).

In conclusion, we detected relatively high (micromolar) concentrations of 23 plasma flavanone and phenolic metabolites 5 h after orange juice intake but observed no acute changes in simultaneously assessed cardiovascular risk biomarkers, relative to a sugar and vitamin C–matched control. It is clear that the acute effects of flavanone consumption remain in question, and further evidence is required to establish the exact contribution of flavanones in comparison with other potential bioactives in citrus fruit (including vitamin C), the effect of the food matrix, the contribution of flavanone-derived phenolic metabolites, and, ultimately, the cardiovascular response to longer-term flavanone and phenolic metabolite exposure.

## Supplementary Material

Supplemental data
