# Supplementary material for: Orange juice–derived flavanone and phenolic metabolites do not acutely affect cardiovascular risk biomarkers: a randomized, placebo-controlled, crossover trial in men at moderate risk of cardiovascular disease1
Source: Am J Clin Nutr. 2015 Mar 18;101(5):931–8. doi: 10.3945/ajcn.114.104364 (PMC4409690; doi:10.3945/ajcn.114.104364)
Supplement: Supplemental data [file supp_101_5_931__index.html]

Supplemental data 

# Orange juice–derived flavanone and phenolic metabolites do not acutely affect cardiovascular risk biomarkers: a randomized, placebo-controlled, crossover trial in men at moderate risk of cardiovascular disease

## Supplemental data

**Files in this Data Supplement:**

- Supplemental data - Figures 1-3 and Tables 1 and 2
