# Supplementary material for: Orange juice–derived flavanone and phenolic metabolites do not acutely affect cardiovascular risk biomarkers: a randomized, placebo-controlled, crossover trial in men at moderate risk of cardiovascular disease1
Source: Am J Clin Nutr. 2015 Mar 18;101(5):931–8. doi: 10.3945/ajcn.114.104364 (PMC4409690; doi:10.3945/ajcn.114.104364)
Supplement: Supplemental data [file 114.104364_ajcn104364SupplementaryData1.docx]

|  |
| --- |
| **Supplemental Figure 1.** Chromatograms of orange juice supernatant (A) pellet (B) and hesperidin supplement (C). Compounds were identified based on retention time and absorbance spectrum, respectively: 1, vitamin C (1.6 min; λ_max_ 250 nm); 2, taxifolin (7.7 min; λ_max_ 284 nm, i.e. chromatography standard); 3, narirutin (9.1 min; λ_max_ 284 nm); 4, rutin (9.2 min; λ_max_ 350 nm, i.e. extraction efficiency standard) and 5, hesperidin (10.2 min; λ_max_ 284 nm) |

|  |
| --- |
| **Supplemental Figure 2. Screening strategy for the identification of orange juice derived flavanone and phenolic metabolites.**  ^1^Pure compounds were commercially available or synthesized.  ^2^Pure compounds were not commercially available and identification was based on known transitions.  ^3^Collected 5 h after the orange juice intervention and pooled from n = 13 participants.  ^4^For 9 compounds, concentrations were not significantly different 5 h after the orange juice intervention compared to the control intervention and 4 compounds were below the limit of detection.  ^5^54 phenolics were: 2,4-dihydroxybenzaldehyde, 2,4-dihydroxybenzoic acid, 2,4-dihydroxycinnamic acid, 2,3-dihydroxybenzoic acid, 2-hydroxy,4-methoxybenzaldehyde, 2-hydroxy,4-methoxybenzoic acid, 2-methoxybenzoic acid, 3,4-dihydroxybenzaldehyde, 3,4-dihydroxyhydrocinnamic acid, 3,4-dihydroxyphenylacetic acid, 3,4-dimethoxybenzoic acid methyl ester, 3,5-dihydroxybenzoic acid, 3-hydroxybenzoic acid, 3-hydroxycinnamic acid, 3-hydroxyphenylacetic acid, 3-methoxybenzoic acid, 3-methoxycinnamic acid, 3-methylhippuric acid, 3-methylgallic acid, 4-hydroxybenzaldehyde, 4-hydroxybenzoic acid, 4-hydroxybenzyl alcohol, 4-hydroxyphenylacetic acid, 4-methoxybenzaldehyde, 4-methoxybenzoic acid, 4-methoxycinnamic acid, 4-methylhippuric acid, 4-valerolactone, 5-oxo-5-phenylvaleric acid, 5-phenylvaleric acid, 6-methoxysalicyclic acid, alpha-hydroxyhippuric acid, apocynin, caffeic acid, dihydroferulic acid, ferulic acid, gallic acid, hippuric acid, homovanillic acid, hydrocinnamic acid, isoferulic acid, isovanillic acid, methyl 3,4,5-trihydroxybenzoate, methyl 3,4-dihydroxybenzoate, methyl vanillate, p-coumaric acid, phloretic acid, phloroglucinol aldehyde, protocatechuic acid, salicyclic acid, sinapic acid, syringic acid, vanillic acid, and hydroxyhippuric acid. |

|  |  |  |  |  |  |  |  |  |  |  |
| --- | --- | --- | --- | --- | --- | --- | --- | --- | --- | --- |
| **Metabolite** | **sMRM mode** | **RT**  **(min)** | **LOD**  **(nmol/L)** | **R^2^** | **Precursor ion (m/z)** | **MS/MS fragments**  **(m/z)** | **DP**  **(V)** | **EP**  **(V)** | **CE**  **(V)** | **CXP**  **(V)** |
| Flavanone aglycones: |  |  |  |  |  |  |  |  |  |  |
| Naringenin | - | 25.6 | 0.1 | 1.000 | 271 | 177, 151*, 119, 107 | -57 | -5 | -25, -25, -25, -45 | -3 |
| Hesperetin | - | 25.7 | 0.1 | 0.998 | 301 | 286, 242, 164* | -59 | -5 | -27, -27, -35 | -1 |
| Flavanone phase II metabolites: |  |  |  |  |  |  |  |  |  |  |
| Hesperetin-diglucuronide^1^ | - | 19.5 | 2.5 | N/A | 653 | 477*, 301, 286, 176 | -86 | -5 | -30 | -7 |
| Hesperetin-diglucuronide^1^ | - | 23.0 | 2.1 | N/A | 653 | 477*, 301, 286, 176 | -86 | -5 | -30 | -7 |
| Hesperetin-glucuronide^1^ | - | 24.2 | 4.1 | N/A | 477 | 301*,286, 257, 242 | -86 | -5 | -30 | -7 |
| Hesperetin-glucuronide^1^ | - | 24.8 | 1.0 | N/A | 477 | 301*,286, 257, 242 | -86 | -5 | -30 | -7 |
| Naringenin-7-glucuronide | - | 24.0 | 0.2 | 0.998 | 447 | 271*, 176, 151 | -41 | -5 | -27, -22, -42, | 0 |
| Naringenin-glucuronide^1^ | - | 24.3 | 1.0 | N/A | 447 | 271*, 176, 151 | -41 | -5 | -27, -22, -42, | 0 |
| Phenolic metabolites: |  |  |  |  |  |  |  |  |  |  |
| Benzoic acid-4-glucuronide | - | 3.1 | 0.6 | 0.998 | 313 | 175, 137, 113, 93* | -54 | -7 | -15, -20, -20, -54 | -2 |
| Hydroxyhippuric acid^1, 2^ | - | 4.5 | 4.1 | N/A | 194 | 150, 93*, 121 | -39 | -5 | -20, -27, -38 | -2 |
| 3-hydroxyhippuric acid | - | 5 | 2.4 | 1.000 | 194 | 150*, 93, 121 | -39 | -5 | -20, -27, -38 | -2 |
| Isovanillic acid-3-glucuronide | - | 5.2 | 4.1 | 0.996 | 343 | 152, 113, 167*, 175, 108 | -60 | -4 | -40, -20, -20, -15, -55, | -2 |
| Vanillic acid-4-glucuronide | - | 6.5 | 5.1 | 0.990 | 343 | 152, 113, 167*, 175, 108 | -60 | -4 | -40, -20, -20, -15, -55, | -2 |
| Hippuric acid | - | 7 | 778.5 | 0.997 | 178 | 77, 132, 134* | -40 | -3 | -22, -22, -18 | 0 |
| Iso/vanillic acid-glucuronide isomer^1, 2^ | - | 7 | 4.6 | N/A | 343 | 152, 113, 167*, 175, 108 | -60 | -4 | -40, -20, -20, -15, -55, | -2 |
| 4-hydroxybenzoic acid | - | 7.1 | 18.9 | 1.000 | 137 | 65, 75, 93* | -35 | -5 | -44, -50, -20 | -2 |
| 4-hydroxyphenylacetic acid | - | 7.1 | 148.5 | 0.994 | 151 | 123, 93, 79, 107* | -50 | -7 | -25, -25, -25, -17 | -2 |
| 3-hydroxyphenylacetic acid | - | 8.3 | 7.9 | 0.994 | 151 | 121, 107*, 92.9, 65 | -26 | -7 | -13, -16, -12, -34 | 0 |
| Isovanillic acid | + | 9.9 | 13.9 | 0.997 | 169 | 151, 125, 65, 93* | 27 | 6 | 19, 12, 34, 19 | 6 |
| Vanillic acid | + | 10.3 | 9.2 | 0.998 | 169 | 151, 125, 65, 93* | 27 | 6 | 19, 12, 34, 19 | 6 |
| Iso/ferulic acid-glucuronide^1, 2^ | - | 10.2 | 2.8 | N/A | 369 | 193*, 113, 134, 178 | -28 | -9 | -23, -23, -23, -18 | 0 |
| Dihydroferulic acid-4-glucuronide^1, 2^ | - | 11.5 | 28.9 | N/A | 371 | 151, 195*, 113, 121, 136 | -40 | -8 | -18, -35, -35, -35, -20 | 0 |
| Dihydroferulic acid | - | 17.1 | 5.4 | 1.000 | 195 | 151, 136*, 119, 121, 149 | -40 | -8 | -18, -20, -16, -35, -18, | 0 |
| *sMRM transitions used for quantification.  ^1^Site of conjugation could not be ascertained as identification was based on known transitions where pure standards for isomers were not available or separation of isomers was chromatographically not possible.  ^2^Putatively identified compound which could have different isomeric configuration.  Abbreviations: sMRM, scheduled multiple reaction monitoring; RT, Retention time; LOD, limit of detection (Signal/Noise = 3); R^2^, linear regression coefficient of standard curve; N/A, putative metabolite, i.e. no analytical standard was available to make standard curve; MS/MS, tandem mass spectrometry; DP, declustering potential; EP, entrance potential; CE, collision energy; CXP, collision exit potential. | | | | | | | | | | |

**Supplemental Table 1. Characteristics and parameters for each metabolite assessed.**

|  |
| --- |
| **Supplemental Figure 3: Typical HPLC-electron ionisation-tandem mass spectrometry trace of flavanone metabolites in plasma extracts collected 5 h after participants consumed the orange juice intervention.** Only highest sMRM transitions are displayed for each peak. Bars below the time axis represent number of scheduled multiple reaction monitoring transitions per compound with a window length of 2 min. (Green, negative ionization mode; purple, positive ionization mode; light blue, internal standard.) 1, Benzoic acid-4-glucuronide; 2, hydroxyhippuric acid; 3, 3-hydroxyhippuric acid; 4, isovanillic acid-3-glucuronide; 5, vanillic acid-4-glucuronide; 6, hippuric acid (reached 3.2e5 cps); 7, isomer of vanillic acid-glucuronide or isovanillic acid-glucuronide; 8, 4-hydroxyphenylacetic acid; 9, 4-hydroxybenzoic acid; 10, 3-hydroxyphenylacetic acid; 11, isovanillic acid; 12, isoferulic or ferulic acid-glucuronide; 13, vanillic acid; 14, dihydroferulic acid-4-glucuronide; 15, dihydroferulic acid; 16, hesperetin-diglucuronide; IS, taxifolin (reached 7.0e4 cps); 17, hesperetin-diglucuronide; 18, naringenin-7-glucuronide (reached 7.6e4 cps); 19, hesperetin-glucuronide; 20, naringenin-glucuronide; 21, hesperetin-glucuronide; 22, naringenin; 23, hesperetin. |

**Supplemental Table 2. Chemical structure of identified flavanone and phenolic metabolites.**

|  |  |  |  |  |
| --- | --- | --- | --- | --- |
| **Chemical structure** | **#^1^** | **Common Name** | **IUPAC Name** | **Configuration^2^** |
|  | 23 | hesperetin | (2S)-5,7-dihydroxy-2-(3-hydroxy-4-methoxyphenyl)-2,3-dihydrochromen-4-one | R = OH |
|  | 19  &  21 | hesperetin-glucuronide^3^ | (2S,3S,4S,5R,6S)-3,4,5-trihydroxy-6-[[(2S)-5-hydroxy-2-(3-hydroxy-4-methoxyphenyl)-4-oxo-2,3-dihydrochromen-7-yl]oxy]oxane-2-carboxylic acid ^4^ | R_1_, R_2_ or R_3_ = O-GlcA |
|  | 16  &  17 | hesperetin-diglucuronide^3^ | (2S,3S,4S,5R,6S)-6-[5-(7-{[(2S,3R,4S,5S,6S)-6-carboxy-3,4,5-trihydroxyoxan-2-yl]oxy}-5-hydroxy-4-oxo-3,4-dihydro-2H-1-benzopyran-2-yl)-2-methoxyphenoxy]-3,4,5-trihydroxyoxane-2-carboxylic acid ^5^ | R_1&2_, R_1&3_ or R_2&3_ = O-GlcA |
|  | 22 | naringenin | 5,7-dihydroxy-2-(4-hydroxyphenyl)-2,3-dihydrochromen-4-one | R = OH |
|  | 18 | naringenin-7-glucuronide | 3,4,5-trihydroxy-6-{[5-hydroxy-2-(4-hydroxyphenyl)-4-oxo-3,4-dihydro-2H-1-benzopyran-7-yl]oxy}oxane-2-carboxylic acid | R_1_ = O-GlcA |
|  | 20 | naringenin-glucuronide^3^ | (3S,5R,6S)-6-[4-(5,7-dihydroxy-4-oxo-3,4-dihydro-2H-1-benzopyran-2-yl)phenoxy]-3,4,5-trihydroxyoxane-2-carboxylic acid ^6^ | R_2_ or R_3_ = O-GlcA |
|  | 6 | hippuric acid | 2-(phenylformamido)acetic acid | R = H |
|  | 3 | 3-hydroxyhippuric acid | 2-[(3-hydroxyphenyl)formamido]acetic acid | R_3_ = OH |
|  | 2 | hydroxyhippuric acid^3^ | 2-[(4-hydroxyphenyl)formamido]acetic acid ^7^ | R_1_, R_2_, R_4_ or R_5_ = OH |

**Supplemental Table 2.** (continued)

|  |  |  |  |  |
| --- | --- | --- | --- | --- |
| **Chemical structure** | **#^1^** | **Common Name** | **IUPAC Name** | **Configuration^2^** |
|  | 12 | ferulic acid-4-glucuronide^3^ | (2S,3S,4S,5R,6S)-6-{4-[(1E)-2-carboxyeth-1-en-1-yl]-2-methoxyphenoxy}-3,4,5-trihydroxyoxane-2-carboxylic acid | R_1_ = O-glucuronide,  R_2_ = CH_3_ |
|  |  | isoferulic acid-3-glucuronide^3^ | (2S,3S,4S,5R,6S)-6-{5-[(1E)-2-carboxyeth-1-en-1-yl]-2-methoxyphenoxy}-3,4,5-trihydroxyoxane-2-carboxylic acid | R_1_ = CH_3_,  R_2_ = O-glucuronide |
|  | 15 | dihydroferulic acid | 3-(4-hydroxy-3-methoxyphenyl)propanoic acid | R = OH |
|  | 14 | dihydroferulic acid-4-  glucuronide | (2S,3S,4S,5R,6S)-6-[4-(2-carboxyethyl)-2-methoxyphenoxy]-3,4,5-trihydroxyoxane-2-carboxylic acid | R = O-glucuronide |
|  | 8 | 4-hydroxyphenylacetic acid | 2-(4-hydroxyphenyl)acetic acid | R_2_ = OH |
|  | 10 | 3-hydroxyphenylacetic acid | 2-(3-hydroxyphenyl)acetic acid | R_1_ = OH |
|  | 13 | vanillic acid | 4-hydroxy-3-methoxybenzoic acid | R_1_ = OCH_3_, R_2_ = OH |
|  | 5 | vanillic acid-4-glucuronide | (2S,3S,4R,5R,6S)-6-(4-carboxy-2-methoxyphenoxy)-3,4-dihydroxy-5-methyloxane-2-carboxylic acid | R_1_ = OCH_3,_  R_2_ = O-glucuronide |
|  | 11 | isovanillic acid | 3-hydroxy-4-methoxybenzoic acid | R_1_ = OH, R_2_ = OCH_3_ |
|  | 4 | isovanillic acid-3-glucuronide | (2S,3S,4S,5R,6S)-6-(5-carboxy-2-methoxyphenoxy)-3,4-dihydroxy-4-methyloxane-2-carboxylic acid | R_1_ = O-glucuronide,  R_2_ = OCH_3_ |
|  | 7 | isomer of vanillic acid-glucuronide or isovanillic acid-glucuronide | (2S,3S,4S,5R,6S)-3,4,5-trihydroxy-6-((3-hydroxy-4-methoxybenzoyl)oxy)tetrahydro-2H-pyran-2-carboxylic acid | R_1_ = OCH_3_,R_2_ = OH,  R_3_ = O-glucuronide |
|  | 9 | 4-hydroxy-benzoic acid | 4-hydroxy-benzoic acid | R_2_ = OH |
|  | 1 | benzoic acid-4-glucuronide | (2S,3S,4S,5R,6S)-6-(benzoyloxy)-3,4,5-trihydroxyoxane-2-carboxylic acid | R_2_ = O-glucuronide |
| ^1^ Number refers to peak in Supplemental Figure 3.  ^2^ R = H, unless stated otherwise.  ^3^ Site of conjugation could not be ascertained as identification was based on known transitions where pure standards for isomers were not available or separation of isomers was chromatographically not possible.  ^4^ IUPAC name for hesperetin-7-glucuronide; however, metabolite isomer hesperetin-3’-glucuronide [IUPAC name: (2S,3S,4S,5R,6S)-6-(5-((S)-5,7-dihydroxy-4-oxochroman-2-yl)-2-methoxyphenoxy)-3,4,5-trihydroxytetrahydro-2H-pyran-2-carboxylic acid] or hesperetin-5-glucuronide [IUPAC name: (2S,3S,4S,5R,6S)-3,4,5-trihydroxy-6-(((S)-7-hydroxy-2-(3-hydroxy-4-methoxyphenyl)-4-oxochroman-5-yl)oxy)tetrahydro-2H-pyran-2-carboxylic acid] could also exist.  ^5^ IUPAC name for hesperetin-7,3’-glucuronide; however, metabolite isomer hesperetin-5,3’-glucuronide [IUPAC name: (2S,3S,4S,5R,6S)-6-(((S)-2-(3-(((2S,3R,4S,5S,6S)-6-carboxy-3,4,5-trihydroxytetrahydro-2H-pyran-2-yl)oxy)-4-methoxyphenyl)-7-hydroxy-4-oxochroman-5-yl)oxy)-3,4,5-trihydroxytetrahydro-2H-pyran-2-carboxylic acid] or hesperetin-7,5-glucuronide [IUPAC name: (2S,2'S,3S,3'S,4S,4'S,5R,5'R,6S,6'S)-6,6'-(((S)-2-(3-hydroxy-4-methoxyphenyl)-4-oxochromane-5,7-diyl)bis(oxy))bis(3,4,5-trihydroxytetrahydro-2H-pyran-2-carboxylic acid)] could also exist.  ^6^ IUPAC name for naringenin-4’-glucuronide; however, metabolite isomer naringenin-5-glucuronide [IUPAC name: (2S,3S,4S,5R,6S)-3,4,5-trihydroxy-6-{[7-hydroxy-2-(4-hydroxyphenyl)-4-oxo-3,4-dihydro-2H-1-benzopyran-5-yl]oxy}oxane-2-carboxylic acid] could also exist.  ^7^ IUPAC name for 4-hydroxyhippuric acid; however, metabolite isomer 2-hydroxyhippuric acid [IUPAC name: 2-[(2-hydroxyphenyl)formamido]acetic acid], 5-hydroxyhippuric acid [IUPAC name: 2-[(5-hydroxyphenyl)formamido]acetic acid] or 6-hydroxyhippuric acid [IUPAC name: 2-[(6-hydroxyphenyl)formamido]acetic acid] could also exist. | | | | |
